# Cranial Bone Repair and Regeneration After Trauma: Forensic and Clinical Medico-Legal Consequences

**DOI:** 10.3390/bioengineering12090915

**Published:** 2025-08-26

**Authors:** Sorin Hostiuc, Ionuț Negoi, Veronica Ciocan

**Affiliations:** 1 Department of Legal Medicine and Bioethics, Carol Davila University of Medicine and Pharmacy, 050474 Bucharest, Romania; 2 Department of Surgery, Carol Davila University of Medicine and Pharmacy, 050474 Bucharest, Romania; negoiionut@gmail.com; 3 Department of Forensic Medicine, Bioethics, Medical Ethics and Medical Law, University of Medicine and Pharmacy, 300041 Timisoara, Romania; veronica.luta@gmail.com

**Keywords:** bone regeneration, medical malpractice, forensic identification, cranial bone defects, traumatic brain injury

## Abstract

Cranial bone defects caused by trauma present significant clinical challenges but also difficulties in their forensic analysis. The complexity of cranial anatomy, limited vascularization, and proximity to neural structures complicate natural bone regeneration, often requiring surgical intervention and the use of complex materials and techniques. This review aims to identify relevant data for forensic analysis regarding bone regeneration after trauma, with an emphasis on the materials used and their interpretation in medico-legal contexts. It moves beyond a simple clinical perspective, providing a detailed medico-legal analysis of cranial bone repair and regeneration after trauma. This review aims to give a comprehensive analysis of the forensic and medico-legal consequences associated with cranial reconstruction using autogenic, allogenic, xenogenic, and synthetic materials. It gives a pioneering focus regarding an understudied but critical aspect of forensic and legal medicine, both to postmortem and to clinical elements. By detailing the unique radiographic signatures and physical characteristics of various reconstruction materials, we provide the specialists with a go-to material for the interpretation of these materials in forensic contexts. Furthermore, we will provide a detailed analysis of medico-legal risks, mainly those associated with malpractice claims, focusing our attention on the process of informed consent but also the management and interpretation of surgery-related complications.

## 1. Introduction

Cranial bone defects caused by trauma pose a significant global health challenge. A recent Global Burden of Disease study found that there were 20.837 million new traumatic brain injury cases worldwide, with the highest rates being found in Central and Eastern Europe and the Middle East. Among them, falls were the leading cause overall and in older adults (above 65), while traffic-related injuries were most often found in adolescents and young adults [1]. Combat-related cranial injuries are a particularly challenging subset, with wars in Iraq, Afghanistan, and, more recently, Ukraine and the Middle East. They are associated with increased survival rates due to advanced body armor and battlefield medicine and a particular traumatic mechanism, many of which are caused by explosive devices, leading to a particularly high prevalence of cranial defects requiring reconstruction [2,3,4,5].

The impact of TBI on the patient’s quality of life extends beyond the immediate physical injury and should be evaluated multidimensionally, including neurological consequences, psychological effects, and the social dimension [6]. Neurological and cognitive sequelae are the most devastating aspects of cranial trauma, with almost two-thirds of patients with moderate to severe TBI reporting long-term issues pertaining to cognitive functioning, including memory, attention, and the processing speed of executive functioning [7].

Functional independence may be significantly altered after TBIs, including, among others, the capacity to live independently, form new relationships, engage in hobbies, and pursue a successful career [6]. A specific complication associated with cranial defects is the syndrome of the trephined. This condition is caused by the pressure gradient between atmospheric pressure and subatmospheric intracranial pressure, resulting in the compression of the underlying brain matter [8,9]. It leads to headaches, dizziness, altered behaviors, and difficulties in coordination and daily activities. From a psychological perspective, cranial defects may cause significant distress, as they can visibly alter the shape of the head, leading to decreased confidence or social withdrawal [10].

We will examine the way the choice of material, which ranges from traditional autologous bone grafts to advanced synthetic alternatives such as ceramics, metals, and polymers, is able to create distinct, invaluable, and interpretable data for criminal investigations. Even if autologous bone remains the gold standard in clinical environments due to its superior osteointegration and biocompatibility, its usage is often limited to small- or medium-sized defects. In larger defects, the surgeons must often use a variety of allogenic, xenogenic, or synthetic materials such as calcium phosphate cements, titanium mesh, or polyetheretherketone (PEEK) implants, each posing unique mechanical and radiographic properties that are able to influence the medico-legal analysis directly. For example, a PEEK implant, because it is radiolucent, may obscure the underlying bone healing on an X-ray. In contrast, titanium implants could lead to severe imaging artifacts. Still, their particular structural characteristics, and the fact that they are serialized, could prove to be a powerful tool for identification in forensic, postmortem contexts. From a clinical medico-legal point of view, the selection and application of these materials may also have significant malpractice-related consequences. We will analyze, in the last part of this article, key issues in this area, including the management of the informed consent process and the proper management of surgery-related complications, such as those related to infection and implant failure, both causing increased costs for patients due to revision surgery and, subsequently, an increased legal risk.

## 2. Classification and Assessment of Cranial Defects

Cranial bone defects are highly heterogeneous and complex, requiring a robust classification system that addresses multiple dimensional parameters, such as the defect size, anatomical location, and tissues involved. For example, Sahoo et al. classified the defects based on their size and anatomical location. Depending on the size, they divided the defects into small (<25 cm^2)^, medium (25–100 cm^2)^, and large (>100 cm^2)^. Depending on the anatomical location, they were classified as simple, involving a single bone; compound, involving two adjoining bones; complex, involving three or more adjoining bones; and complicated, which crossed the midline or were bilateral. They also recommended using simple letters to depict the location (O, occipital; T, temporal; F, frontal; P, parietal; and SZ, subzygomatic) [11]. Uygur et al. considered that medium and moderate cranial defects should be extended up to 200 cm^2^, and large defects should be considered those above this threshold [12].

Small cranial defects, typically measuring less than 25 cm^2^, are the most favorable category for reconstruction, with an excellent prognosis. The biological rationale for this size limitation is the effective vascular penetration distance from the peripheral margins of the bone. These defects can easily be vascularized from the surrounding bone edges, and the preferred approach is to use autogenous calvarial grafts due to their superior biocompatibility, integration characteristics, and osteogenic potential [11,12]. Medium-sized cranial defects present intermediate reconstructive challenges, requiring more sophisticated approaches. They usually exceed the effective vascular penetration distance from the edges of the bone and include split calvarial grafts, typically for those with an area well below 100 cm^2^, allogenic bone grafts, or synthetic materials [11,13]. Large cranial defects are the most challenging reconstructive scenarios, usually requiring the use of synthetic materials, such as polymethyl methacrylate, titanium mesh systems, or a combination of multiple materials or complex reconstructive procedures [11,12].

The anatomical location of cranial defects significantly influences the healing potential, reconstructive options, and overall clinical outcomes. For example, parietal bone defects, a very common location for traumatic defects, have favorable healing characteristics owing to an abundant blood supply and minimal interference from adjacent structures [14,15]. Frontal bone defects, on the other hand, pose unique challenges due to the proximity of the frontal sinuses and the aesthetic importance of this region, with even minimal alterations of the normal structure leading to a significant aesthetic effect [16,17,18]. A brief overview, summarized on the basis of [13,14,15,19,20], is presented in Table 1.

The number of affected bones is another factor that potentially complicates the analysis. Bone defects affecting a single bone without compromising adjacent structures are, as a general rule, the easiest to reconstruct, with minimal changes to the body scheme [11,13]. Complex defects involving three or more adjoining bones, or bilateral defects, require extended surgical procedures and highly sophisticated reconstructive planning [13].

Cranial defects are usually not isolated and are associated with a variable degree of soft tissue involvement, including the scalp, periosteum, dura mater, or even the brain matter [21,22,23,24,25]. Scalp involvement necessitates an assessment of both the quantity of lost tissue and the quality of the remaining soft tissue coverage [22]. Defects with intact, well-vascularized scalp coverage have more favorable outcomes and a reduced risk of complications than those requiring simultaneous soft tissue reconstruction [22,26,27,28,29,30]. The presence of an intact dura mater protects the underlying neural structures and promotes bone healing through the release of osteogenic factors [31,32,33,34,35]. The presence of dural defects requires immediate repair to reduce complications.

A sufficient blood supply is crucial for bone regeneration [28,36]. Compromised vascular supply is one of the primary limitations of cranial bone healing [37]. Periosteal vascular network damage has been shown to significantly decrease the healing potential and may require the use of vascularized reconstruction techniques [29,38,39].

The concept of critical-size defects (CSDs) is a fundamental issue related to cranial bone repair and regeneration. Schmitz and Hollinger initially defined it as the smallest intraosseous wound in a particular bone and species of animal that will not heal spontaneously during its lifetime [30]. Currently, it is understood as a cranial defect that cannot be healed without surgical intervention, with a variable size depending on the anatomical location and individual patient-related factors. A recent meta-analysis on rodent models suggested that a calvarial defect exceeding 5.0 mm should be considered a CSD; however, the heterogeneity of the included studies was high [40]. In dogs, 20.0 mm was considered the critical threshold [30]. In humans, there is no clearly defined CSD value, as a study to properly evaluate it would likely breach numerous ethical recommendations. In addition, extrapolation from animal models is not recommended, as the bone structure and capacity for bone regeneration differ significantly [41]. Although a human calvarial defect exceeding 2–2.5 cm is considered incapable of spontaneous healing, these values have not been proven through a high-level scientific approach [42,43,44]. The unique anatomical features of the cranial bone structure present specific challenges for regenerative approaches, which differ significantly from long bone repair. The human cranium contains 22 bones, divided primarily into two main regions: the neurocranium, which protects the brain and sensory organs, and the viscerocranium, which supports the facial structure. This complex architecture encompasses bones with distinct developmental origins, healing characteristics, vascularization, thicknesses, and ratios of cortical versus porous bone, among other features [45]. Cranial bone formation is driven by two distinct ossification processes. The cranial base is primarily formed through endochondral ossification, resulting in a more compact, dense, and less flexible structure. In contrast, the cranial vault is formed chiefly through intramembranous ossification, leading to less strength but increased flexibility, which is further augmented by the presence of lateral sutures, which are pivoted and beveled to allow for a minor degree of movement [46]. Unlike long bones, which have a robust medullary blood supply, cranial bones mainly depend on periosteal and dural circulation. This constrained vascular network, which may be further compromised following TBIs associated with dural tears or periosteal stripping, further decreases regenerative potential [47]. The proximity of the brain to the cranial bones raises concerns about biocompatibility, inflammatory responses, and potential neurological complications associated with the use of allogeneic grafts for bone defect treatment [47]. The biological barriers to natural cranial bone healing are summarized in Table 2.

## 3. Materials Used for Cranial Bone Reconstruction

### 3.1. Autologous Grafts

Autologous grafts are the historical gold standard for cranial bone reconstruction, offering significant advantages over most other materials, including those related to biocompatibility, osteogenic potential, and integration characteristics [51,52,53]. Where donor site availability permits their use, they play a central role in reconstruction, especially for small- and medium-sized defects. The most widely used autologous option for cranial reconstruction is split calvarial grafts, which have as their main advantages increased anatomical compatibility and minimal donor site morbidity compared to using grafts from distant locations. The techniques involved harvesting the outer cortical table while leaving the inner one in situ, thereby creating two usable bone grafts from a single donor site [54,55]. The clinical outcomes were highly favorable. Frautschi, for example, in a series of 41 patients with an average cranial defect of 68 cm^2^, showed a 73% success rate using split calvarial grafts, despite 85% of the patients having significant risk factors. The grafts maintained their thickness over time, with a mean ratio of graft to bicortical donor site thickness of 0.48 for recipient sites [54]. This technique is very versatile, enabling the restoration of continuity across multiple anatomical structures, including the frontal bone, supraorbital ridge, and orbital roof. For example, Papay et al. have shown that sphenoid wing reconstruction using split calvarial grafts yields excellent outcomes, with complete resolution or significantly improved proptosis in all patients and virtually no osteomyelitis or graft loss during an extended follow-up period [56]. It may also be used in pediatric populations, which require special consideration during the ongoing development of cranial bones. For example, Hayeem et al. showed that the split-bone technique for pediatric craniosynostosis has a 100% success rate. None of the patients in a study of 162 participants required reoperation for either resorption or contour irregularities [57]. Alternate autologous harvest sites can be used when calvarial donor sites are inadequate or unavailable. They include sagittal split rib bone grafts, which are helpful for bilateral cranial defects, or in patients with a previous history of infection, precluding calvarial harvesting [58]; iliac crest, which has the advantage of allowing for the harvesting of larger amounts of cancellous bone showing an equivalent effectiveness to cranial bone grafts for certain reconstructions such as orbital or mandible repair [59,60]; or various other bones, with specific uses depending on the type of defect and particular availability [59,61].

### 3.2. Allogenic Materials

Allogenic materials are bone grafts derived from human donors, usually cadavers or brain-dead patients, and are specifically processed and prepared for transplantation. There are three main types of allogenic materials: fresh-frozen bone allografts (FHBA), freeze-dried bone allografts (FDBA), and demineralized bone matrix (DBM). FHBA is preserved through freezing after harvesting; it maintains much of the bone’s structural integrity and some biological properties, providing excellent structural support [57,58,59]. However, it has limited availability and temporal constraints [62,63]. FDBAs undergo a dehydration process that removes water before use while preserving the bone matrix. This approach extends the shelf life and decreases immunogenicity while maintaining the bone’s osteoconductive properties [64,65,66]. DBM is the most widely used allogenic material for cranial reconstruction, offering osteoinductive properties through its retained morphogenic proteins and growth factors. Its processing involves the extraction of mineral content while preserving the organic collagen matrix and associated bone growth factors, which are essential for promoting bone formation [52,67,68,69,70].

### 3.3. Xenogenic Materials

Xenogenic materials are derived from animal sources, usually bovine or porcine. They have been demonstrated to be effective as osteoconductive scaffolds for cranial bone regeneration, offering significant advantages in terms of availability and cost compared to human allografts. Bovine sources are preferred due to their availability in large quantities, their anatomical similarities with human bone structure, and the presence of well-established protocols ensuring their safety and sterility [51,71,72,73]. Porcine-derived xenografts are less often used, even though they seem to have similar efficiency in terms of their cellular response and bone regeneration ratio [72]. Tissue processing includes several stages designed to remove immunogenic components while simultaneously preserving the osteoconductive mineral matrix. It consists of the removal of all organic components through chemical extraction and/or thermal treatment, leaving only the trabecular bone structure [74,75,76]. Bovine hydroxyapatite retains the natural lamellar bone structure after tissue processing, leaving a material with a structure similar to that of the human cancellous bone [73,75].

### 3.4. Bioceramics

Bioceramic materials are a heterogeneous class of synthetic compounds with excellent biocompatibility and osteoconductive properties. They can be engineered to provide specific degradation rates, mechanical properties, or biological responses, thereby optimizing bone formation and osteointegration.

The most extensively studied bioceramic is hydroxyapatite, which has been shown to have excellent long-term outcomes, with extensive osteointegration and a decreased risk of complications [77]. The rate of osteointegration differs widely between studies, but most suggest a 50–75% success rate [78,79,80]. Pure hydroxyapatite has been shown to have some limitations in clinical practice, primarily related to its brittleness, which increases the risk of fracture under impact loading [81]. Carbonate hydroxyapatite, which contains approximately 7.4% carbonate ions, closely resembles human bone, resulting in enhanced osteogenic properties [82]. The inclusion of europium ions is beneficial because it provides luminescent tracking capabilities, enabling the real-time monitoring of scaffold degradation and new bone formation [83]. β-TCP scaffolds exhibit superior bone healing properties, particularly in pediatric studies, with a 79% success rate, defined as the absence of persistent bone gaps after reconstructive procedures for craniosynostosis [84].

Calcium phosphate cements are a type of injectable bioceramic system that provides in situ hardening capabilities, enabling precise defect filling and contouring during reconstruction, and they are associated with excellent biocompatibility and osteoconductive properties [85,86]. Their use has significantly lower wound infection and cerebrospinal fluid leak rates than other materials or methods, such as titanium mesh cranioplasty [85]. When combined with antibiotics, they can be used to address specific challenges, such as infection cases [87]. Long-term studies have shown that these materials may undergo partial resorption and remodeling, limitations that can be overcome with the concomitant use of supportive frameworks, such as titanium meshes [85].

Bioactive glasses are a special type of synthetic material that can form chemical bonds with living bone tissue. They typically contain SiO_2_ as the primary component, along with Na_2_O, CaO, and P_2_O_5_ [88,89,90]. When they come into contact with body fluids, they undergo a series of chemical reactions. The local glass surface releases ions that increase the local pH, creating a favorable environment for bone formation. The silica-rich gel layer on the surface gradually transforms into a hydroxyapatite layer over time, enabling the glass to chemically bond with the surrounding bone tissue. It exhibits a characteristic change in mechanical properties over time, transitioning from brittle (in vitro) to an elastoplastic response 12–24 weeks after implantation [91]. Bioactive glass materials were also shown to facilitate the establishment of a proper blood supply locally by promoting neoangiogenesis [91], while special types of bioactive glasses, such as silococarnotite bioceramics, were shown to promote the osteogenic differentiation of stem cells and enhance critical-sized calvarial defect healing through an improved cellular response [92].

### 3.5. Other Materials

Metallic implants, especially those made of titanium and its alloys, are known to have excellent biocompatibility and mechanical properties [93,94]. Their clinical performance has been evaluated in multiple populations and defect characteristics. For example, Hitoshi et al. demonstrated excellent long-term results using titanium mesh reconstruction, allowing for a return to normal activities, including sports, without complications over an eight-year period [95]. These meshes can be created using traditional methods, intraoperative bending, and the shaping of standard mesh products or modern technologies such as 3D printing, which can create patient-specific implants, resulting in a superior fit and superior aesthetic results [23,96]. For example, di Cosmo et al. conducted a meta-analysis comparing personalized 3D-printed titanium meshes with standard implants for cranial reconstruction, finding that recipients of 3D-printed titanium implants exhibited approximately four times lower complication rates and three times lower infection rates compared to standard implants [97].

Biodegradable polymers are extremely useful in providing temporary mechanical support during healing, ultimately being replaced by natural tissue [98,99]. Three substances are primarily used for cranial applications: polylactic acid (PLA), polycaprolactone (PCL), and poly(lactic-co-glycolic) acid (PLGA), each of which offers specific degradation rates and mechanical properties and can subsequently be optimized for specific clinical scenarios [99,100,101]. The details of the specific uses are presented in Table 3.

Hydrogels can be injected into irregular cranial defects, providing aesthetic repair by conforming to the natural contours of the vault. They exhibit high plasticity and moldability, enabling the reconstruction of complex three-dimensional defects that would be difficult to repair using rigid implant materials [105]. They can also be used as drug delivery systems for antibiotics, bone morphogenetic protein-2, and insulin-like growth factor 1 [106]. Smart hydrogel systems can respond to various physiological conditions, such as pH changes, temperature variations, and enzyme activity, thereby optimizing therapeutic delivery and scaffold degradation [105]. They can be delivered using minimally invasive techniques, such as small incisions and percutaneous approaches, thereby minimizing surgical morbidity and enabling the treatment of defects that might be inaccessible through conventional surgical approaches [107].

## 4. Forensic Consequences of the Materials Used in Cranial Reconstruction

In forensic and legal medicine, the materials used for cranial reconstruction have significant, although understudied, uses in areas such as postmortem identification, human remains analysis, and medical malpractice. As the complexity and variety of materials used in cranial reconstruction continue to expand, forensic pathologists must understand how they can affect identification processes, image interpretation, and evidentiary analysis.

### 4.1. Radiographic Identification and Imaging Artifacts

The presence of cranial reconstruction materials alters the radiographic appearance of the human remains. Different materials produce distinct radiographic signatures, which can aid in identification but also interfere with standard forensic imaging protocols [108,109]. For example, metallic implants are known to cause significant artifacts in both CT and MR imaging [110]. Alumina ceramics containing Yttrium make the material radiopaque, which complicates imaging and future radiation treatments in patients with cranial reconstructions that use them [111]. Kline et al. studied various types of materials used for dental restoration and found that, among the 44 materials studied, artifacts were observed in 13 using MRI and 41 using CT [112]. Titanium implants are known to cause specific susceptibility artifacts, which can obscure anatomical features. In contrast, other materials, such as polyetheretherketone (PEEK), exhibit superior MRI compatibility, resulting in minimal artifacts [113]. MRI leads to signal voids, image distortion, and susceptibility artifacts, especially pronounced in the Gradient Echo sequences, while Turbo Spin Echo sequences have lower artifact rates [113]. The development of sophisticated imaging technologies has mitigated this problem, using complex algorithms [109,110,113,114,115,116].

On the other hand, metallic implants, especially titanium-based systems, have excellent radiographic visibility. This, combined with the uniqueness of each implant, makes them powerful tools for identification through the comparison of antemortem and postmortem radiographs [117,118,119]. For example, Prado et al. described the case of a victim of a gunshot wound found on a highway, whose identity was established by comparing the antemortem X-ray of a disappeared person with the postmortem radiograph of the skull, the identification being made on the basis of the shape of the frontal sinus, the shape and height of the orbits, and a titanium miniplate used for bone fixation [120]. Metallic structures used for osteosynthesis can be identified and used for positive identification even after explantation. Palazzo et al., for example, performed a histological evaluation on 12 corpses that had undergone osteosynthesis intra vitam, some of which still had their implants, while others had their implants removed before death. Using classical histopathology and scanning electron microscopy with energy-dispersive spectroscopy, they found the presence of intra-bone metal particles in the tissues treated by osteosynthesis, even though no radiological signs of their presence were found [121]. They could also aid in the identification of severely burned charred bodies, as some have a very high melting point, retaining their shape even when other bodily structures are completely destroyed. For example, Francesquini et al. described a case in which a positive identification of a severely charred person, with calcinated bones and teeth, was made through the use of a maxilla with an implant/individual fixed metallo-ceramic prosthesis, which retained its shape sufficiently to allow for a positive identification based on antemortem data [122].

The utility of forensic radiological identification is limited by the quality and availability of antemortem records, which are often not readily available or, if available, have difficulties in identifying the probable identity of the victim [41].

The development of artificial intelligence algorithms for analyzing radiographic images shows promise in standardizing implant identification procedures and minimizing the negative effects of artifacts. Automated systems have been shown to achieve accuracy rates of over 90% in identifying cardiac implantable devices from radiographs [123], suggesting that a similar approach could be used for cranial reconstruction materials. The use of advanced algorithms for forensic identification, such as the analysis of geometric cranial patterns on lateral cephalometric radiographs, has an accuracy rate of over 97% [124]. Numerous studies have evaluated the usefulness of machine learning for human identification using cranial features by examining various imaging modalities, including 2D dental radiographs, dental radiographs, panoramic X-rays, CT scans, CBCT, and MRI. Some of the developed tools have achieved over 90% accuracy and can process large datasets rapidly [125]. Their usefulness can be further enhanced by identifying restorative cranial materials and incorporating them into the analysis.

### 4.2. Material-Specific Forensic Signatures

The presence and type of restorative material can significantly impact forensic identification. Autologous bone grafts, even if they are known to integrate completely with the surrounding bone if enough time has passed, are known to still retain subtle morphological and histological changes, which can be detailed through a detailed analysis [121,126]. These changes can also be identified at the site of explantation, further increasing the accuracy of positive identification. Histologically, the donor site undergoes bone remodeling, and even if the harvested area can regenerate, the process may be slow and incomplete, especially in older patients. If an infection occurs at the donor site or in patients with significant comorbidities, the process may be further hindered [126,127,128]. The incorporation of surgical hardware in grafting procedures, such as fixation plates or screws, may offer additional opportunities for identification through manufacturer-tracking systems [129,130].

Metallic implants have the most robust forensic signatures due to their distinctive design features, manufacturer markings, and serial numbers. These signatures have allowed for the successful identification of unknown human remains, even after extreme environmental exposure [121,131,132]. Manufacturing tracking systems are required by regulatory agencies, enabling the creation of comprehensive databases that facilitate the identification of device origins, distribution chains, and patient records.

Synthetic materials, such as Polymethylmethacrylate (PMMA) and PEEK, exhibit varying degrees of thermal and chemical resistance, which affects their forensic utility [133,134]. Another issue that must be considered is that these materials are sometimes detached due to decreased bone density in the peri-implant area, especially in recent implants. Therefore, restorative materials should be thoroughly researched in the surrounding areas, especially when the body is exposed to extreme conditions, such as high temperatures [133].

Calcium phosphate cements, owing to their variable resorption patterns and potential for complete biological replacement, may limit their long-term identification utility [135,136]. Bioactive glass is also known to undergo progressive transformation through dissolution and precipitation, creating a time-dependent forensic signature; however, it leads to the de novo formation of well-vascularised bone tissue with a morphology that is practically identical to the native bone over extended periods of time [88,137], so its usefulness for identification is decreased, but it may be used to estimate postmortem intervals or implantation timelines. This is similar to other materials, such as calcium phosphate bioceramics, including monetite-based composites and beta-tricalcium phosphate, which are known to have volumetric decreases of approximately 8% in the first year, with a plateau thereafter, and annual degradation rates ranging from 54.6% to 90.5%, depending on the scaffold design or additives [138,139,140]. Studies on polymer-based scaffolds, such as those made from PCL and PGLA, have shown that increasing the number of bioactive additives or altering the fabrication methods may accelerate or decelerate degradation [141]. Ultimately, for proper use in forensic contexts, extensive data on these issues should be built in updated databases, with free availability for any researcher in the field.

### 4.3. Thermal Resistance

The best thermal resistance is found in titanium implants, which retain their structural integrity at temperatures exceeding 1000 °C. Most studies conducted in this regard focused on dental titanium implants, which were shown to remain completely identifiable even at temperatures at which soft and hard tissues were destroyed [133,134,142]. Additionally, the preservation of serial numbers and manufacturer markings provides reliable identification markers for cases involving extreme thermal exposure. Various metallic alloy compositions exhibit different responses to electromagnetic heating. For instance, stainless steel implants demonstrate greater susceptibility than titanium alloys. This feature may aid in the reconstruction of fire scenarios or electromagnetic exposure events [143].

Ceramic materials have an intermediate thermal resistance, retaining their structural integrity at moderate temperatures while undergoing crystalline changes at higher temperatures, which can be detected using specialized analytical techniques. The identification of specific degradation products or phase changes may provide useful data on the peak temperatures reached during thermal exposure [144,145,146,147,148].

### 4.4. Postmortem Material Degradation and Environmental Effects

Environmental factors, including pH, moisture, soil composition, and microbial activity, can significantly influence degradation rates, particularly for biological materials. Corrosion and oxidation processes can alter metallic implants, creating unique features that reflect both environmental conditions and the duration of exposure [121]. The metals released from osteosynthesis materials are identifiable both in the bone, even after explantation, and in the soil, especially in the absence of explantation [149,150].

The chemical degradation of polymer materials can also occur through specific pathways that can be characterized using advanced analytical techniques. There are, however, interposing factors that limit this analysis, including the materials from which the clothing of the victims is made [151] or surrounding materials of interest, such as accelerators used in fires and the structures in which the body is found (car, grave, etc.) [152].

## 5. Medical Malpractice Related to Cranial Reconstruction Materials

Neurosurgery consistently ranks among the medical specialties with the highest malpractice exposure, and cranial procedures represent a significant proportion of all claims. In the United States, 19% [153] of all neurosurgeons face a medical malpractice suit annually, leading to an average indemnity paid of almost USD 450,000 [154,155]. Interestingly, lawsuits were not only generated by the appearance of significant complications such as infections but also by patients who had not seen the benefits they were hoping for [156]. In craniofacial surgery, a retrospective review conducted between 1981 and 2024 using the Westlaw and LexisNexis databases identified the most frequently litigated craniofacial procedures as palatoplasty, followed by cranial vault reconstruction, facial fracture repair, and mandibular reconstruction. Less than one-third resulted in plaintiff verdicts or financial settlements, with an average payout of $2.5 million [156]. In pediatric neurosurgery, cranioplasty is a significant subset of malpractice claims, as revealed by an extensive study conducted in Germany [157].

As shown above, one of the significant risk factors for malpractice claims is a lack of understanding on the part of the patient or their legal representative of the benefits and burdens associated with these procedures [156]. This suggests a significant information-related issue, with many patients unable to grasp or understand the information. A systematic review of informed consent in neurosurgery found that almost half of the patients failed to recall relevant data provided to them, and the average number of risks remembered was only 5 out of 32 [158]. The material selection process requires extensive disclosure regarding different reconstruction options; patients must be informed about the type of material (autologous, allogenic, xenogenic, or synthetic), each with a distinct risk profile, success rate, and long-term consequence. The choice of materials must be supplemented by comparative discussions on infection rates, mechanical failure potential, aesthetic outcomes, and the potential need for revision surgeries [111,159]. Pediatric cases especially must receive additional information about growth-related considerations, long-term developmental impact, and material selection appropriate for extending skulls [160]. Parents or legal representatives must clearly understand that certain materials may need to be replaced as the child grows, while others may interfere with normal cranial development. The timing of the intervention should also be clearly defined, as a specific type of reconstruction may only be recommended up to or from a particular age [161,162,163,164].

The standard of care in cranial reconstruction is continually evolving, with advancements in materials and techniques being developed and introduced into clinical practice and guidelines each year. For example, traditional approaches using autologous bone grafts have long represented the gold standard; however, the use of synthetic materials often provides better results and a decreased burden, at least in some cases [111,165]. Open cranial vault reconstruction is the standard for craniosynostosis; however, the use of endoscopic procedures has been shown to have superior safety profiles, at least in specialized centers [166].

Different reconstruction materials require adherence to specific protocols and have specific contraindications. For example, PMMA implants have a higher infection rate than autografts, necessitating specific handling protocols [111,167]. Titanium mesh systems have superior outcomes in specific scenarios, including a significantly decreased infection rate; however, they are usually more expensive and require more advanced fabrication techniques [111]. If the patients would need repeated MRI, this technique should be avoided, as it could significantly interfere with MR imaging results [136,168].

Liability may arise from manufacturing defects, design flaws, or inadequate warnings about the limitations and contraindications of various materials. Manufacturing defects include improper sterilization, contamination, dimensional inaccuracies, and material inconsistencies [169]. Design defects encompass flaws in implant conception or material selection, which can create unreasonable risks for patients. This includes the brittle nature of certain ceramic materials, the thermal conductivity of metallic implants, and degradable products from biodegradable polymers [111]. Their effects should be minimized whenever possible, and patients should be thoroughly informed about the potential risks and benefits associated with them.

Infectious complications are the most common cause of cranial reconstruction failure, generating a significant proportion of malpractice claims. The infection rates tend to differ significantly depending on the material, with MPP having the highest rate (almost 11%), while autografts have only a 2% rate [111]. Material-specific infections may require specialized management protocols, including implant removal, extended antibiotic therapy, or delayed reconstruction. Failure to properly recognize implant-related infections can lead to brain abscesses, meningitis, encephalitis, or sepsis, with potentially fatal consequences [170,171,172,173].

The syndrome of the trephined is a specific complication dependent on the size of the cranial defect and the timing of reconstruction, potentially generating legal claims when inadequately addressed [8,9]. Failure to offer timely reconstruction for defects exceeding critical size thresholds may represent substandard care, especially considering the probability of neurological improvement following adequate reconstruction.

Another area prone to malpractice claims is mechanical implant failure and the subsequent need for revision surgery. PMMA implants are particularly susceptible, with a reported fracture rate of 6.7% [168,174]. Even if patients remain asymptomatic, revision surgery is still necessary to restore the implant’s function, as PMMA is unable to undergo bone repair [175]. Overall, the need for revision surgery ranges from 10% to 23% for alloplastic materials, depending on the material, patient factors, or surgical technique [165]. Incorrect information about this risk and the need for multiple revision surgeries could significantly increase the probability of malpractice claims.

Aesthetic outcomes significantly alter the quality of life of patients. Therefore, suboptimal aesthetic results could increase the risk of litigation. Poor aesthetic results can be caused by inadequate contouring, asymmetry, or visible implant edges [176,177,178,179]. These issues have been largely addressed by using patient-specific implant designs; however, older techniques and inappropriate materials are still in use, especially in non-reference centers.

## 6. Concluding Remarks

This review aims to show that the process of cranial bone repair and regeneration is a complex, multidisciplinary field, in which clinical success is increasingly tied to a detailed and profound understanding of materials science, being essential for the application of this knowledge in forensic and legal medicine. Even though autologous grafts remain the gold standard for many cranial repair procedures, the use of allogenic, xenogenic, and synthetic materials is often essential for addressing a wider range of more complex traumatic consequences. The choice of material is not merely a clinical decision; it is a critical variable with long-term implications for a patient’s health and, importantly, for postmortem identification.

A key contribution provided by this paper is its focus on the forensic and medico-legal consequences, an area that has been severely understudied. We have shown that the unique characteristics of each material may be used as a tool for positive identification in forensic investigations. Also, we have emphasized the potentially significant legal risks, especially in regard to medical malpractice and informed consent, which stem directly from the selection and application of these materials.

The forensic and legal evaluation of cranial reconstruction and repair must continue with an interdisciplinary approach. Future research should not only aim to improve the biocompatibility and regenerative potential of materials but also consider their forensic utility from the initial design phase. By fostering collaboration between neurosurgeons, neurologists, material scientists, and also medico-legal and forensic experts, we may ensure that advancements in cranial surgery not only enhance patient outcomes but also provide clearer, more reliable evidence for forensic analysis, ultimately bridging the gap between clinical and medico-legal medicine.

## Figures and Tables

**Table 1:** Location-Based Classification of Cranial Defects.

Location	Description	Reference Point	Reconstruction Approach	Critical Structures	Healing Characteristics	Special Considerations
Anterior Cranial Fossa—Localized	Confined to cribriform plate area	Cribriform plate	Locoregional flaps	Frontal lobes, olfactory apparatus, orbital contents	Variable	Most complex anatomical region [13,14,15,19,20]
Anterior Cranial Fossa—Horizontal	Extended to the orbital roof	Cribriform plate	Free flap reconstruction	Frontal lobes, olfactory apparatus, orbital contents	Variable	Requires free flap due to orbital involvement [13,14,15,19,20]. Autologous grafts are the historical gold standard
Anterior Cranial Fossa—Vertical	Extended to deep sinonasal cavity	Cribriform plate	Locoregional flaps	Frontal lobes, olfactory apparatus, orbital contents	Variable	Deep sinonasal involvement [13,14,15,19,20]
Middle Cranial Fossa—Localized	Confined to the infratemporal fossa	Infratemporal fossa	Locoregional flaps	Carotid artery, internal jugular vein, multiple cranial nerves	Variable	Complex neurovascular anatomy [13,14,15,19]
Middle Cranial Fossa—Horizontal	Involves pterygoid muscles or mandible	Infratemporal fossa	Free flap reconstruction	Carotid artery, internal jugular vein, multiple cranial nerves	Variable	Most extensive reconstruction required [13,14,15]
Middle Cranial Fossa—Vertical	Extended to maxillary sinus or nasopharynx	Infratemporal fossa	Similar to localized defects	Carotid artery, internal jugular vein, multiple cranial nerves	Variable	Maxillary sinus/nasopharynx involvement [13,14,15]
Posterior Cranial Fossa	Occipital region and posterior temporal bone	Occipital region	Minimal intervention, local tissue rearrangement	Well-vascularized soft tissue	Excellent—robust healing capacity	Positioning and access challenges [13,14,15,19]
Frontal Bone	Frontal region defects	Frontal bone	Variable	Frontal sinuses	Variable	Aesthetic importance, sinus proximity [13,14,15]
Parietal Bone	The most common traumatic location	Parietal bone	Variable	Minimal adjacent structures	Excellent (68.4% closure rate)	Robust blood supply, favorable healing [13,14,15]
Fronto-Parietal	Combined frontal-parietal involvement	Frontal-parietal junction	Variable	Frontal sinuses, minimal parietal structures	Moderate (43.7% closure rate)	Lower healing than isolated parietal [13,14,15]
Temporal Bone	Temporal region defects	Temporal bone	Variable	Middle meningeal artery, temporal muscle	Variable	Vascular anatomy considerations [13,14,15]
Occipital Bone	Posterior skull defects	Occipital bone	Local tissue rearrangement	Minimal critical structures	Good	Patient positioning challenges [13,14,15]

**Table 2:** Biological and molecular barriers to natural cranial bone healing (based on [40,47,48,49,50]).

Barrier Category	Specific Limitations	Mechanisms	Clinical Impact
Cellular Barriers	Reduced osteoprogenitor cell density	Periosteal cellular population differs from long bones Altered differentiation potential Disruption of periosteal and endosteal surfaces eliminates osteoblastic precursors	The primary limitation in cranial bone regeneration Dura mater cannot compensate for periosteal loss in significant defects
Vascular Limitations	Inadequate vascularization	No robust medullary circulation system Healing depends entirely on the peripheral blood supply Defects exceeding vascular penetration distance become hypoxic	Critical-sized defects cannot heal naturally Central regions develop fibrous tissue instead of bone
Growth Factor and Cytokine Signaling	Altered expression patterns	Disrupted bone morphogenetic proteins (BMPs) Altered vascular endothelial growth factor (VEGF) Disrupted signaling cascade	Prevents normal sequence of inflammation, proliferation, and remodeling phases
Mechanical Environment	Inadequate mechanical stimulation	Cranial bones experience protective rather than load-bearing stimuli Absence of regular mechanical loading Reduced mechano-biological signals	Limits bone formation and remodeling capacity compared to member’s skeleton
Inflammatory Response	Constrained inflammatory environment	Proximity to brain tissue requires controlled inflammation Excessive responses must be avoided to prevent neurological complications	Limited initial healing phases that typically depend on controlled inflammatory signaling
Age-Related Barriers	Decreased regenerative capacity with aging	Decline in stem cell populations Altered growth factor expression Reduced vascular responsiveness	Progressively greater barriers in older patients (4 mm critical defect size in aged rats vs. 5 mm in young adults)

**Table 3:** Comparison of biodegradable polymers for cranial reconstruction (based on data from [99,100,101,102,103,104]).

Property	PLA (Polylactic Acid)	PCL (Polycaprolactone)	PLGA (Poly(lactic-co-glycolic acid))
Degradation Rate	Moderate	Slow	Rapid
Degradation Timeline	Variable	12–24 months	Variable (controllable via lactide: glycolide ratio)
Mechanical Properties	Variable	Excellent mechanical strength, maintains integrity during healing	Enhanced when in composite form
Biocompatibility	Good	Excellent	Good
Degradation Control	Limited information	Controlled degradation without harmful byproducts	Highly controllable (50:50 ratio provides rapid degradation)
Manufacturing	3D printing-compatible	3D printing-compatible, patient-specific implants possible	3D printing compatible
Composite Applications	Limited information	PCL/β-tricalcium phosphate composites, PCL/PLGA/β-TCP blends	PCL/PLGA/β-TCP scaffolds show enhanced properties

## Data Availability

No new data were created.

## Abbreviations

The following abbreviations are used in this manuscript:

3D	Three-Dimensional
45S5	Specific Bioglass formulation
13-93	Bioactive glass type
β-TCP	Beta-Tricalcium Phosphate
BMP2	Bone Morphogenetic Protein 2
CBCT	Cone-Beam Computed Tomography
CRANIOTOP^®^	Commercial titanium implant system
CSD/CSDs	Critical-Size Defect/Critical-Size Defects
CT	Computed Tomography
kVp	Kilovolt peak
MR/MRI	Magnetic Resonance/Magnetic Resonance Imaging
PCL	Polycaprolactone
PEEK	Polyetheretherketone
PGLA	Poly(glycolide-co-lactide)
PMMA	Polymethylmethacrylate
TBIs	Traumatic Brain Injuries
USD	United States Dollar
VEGF	Vascular Endothelial Growth Factor
